# Clinicopathological Characteristics and Survival Outcomes in Invasive Papillary Carcinoma of the Breast: A SEER Population-Based Study

**DOI:** 10.1038/srep24037

**Published:** 2016-04-07

**Authors:** Yi-Zi Zheng, Xin Hu, Zhi-Ming Shao

**Affiliations:** 1 Department of Breast Surgery, Key Laboratory of Breast Cancer in Shanghai, Fudan University Shanghai Cancer Center, Fudan University, Shanghai 200032, China; 2 Department of Oncology, Shanghai Medical College, Fudan University, Shanghai 200032, China; 3 Institute of Biomedical Science, Fudan University, Shanghai 200032, China

## Abstract

To investigate the clinicopathological characteristics and survival outcomes of invasive papillary carcinoma (IPC), we identified 233,171 female patients in the Surveillance, Epidemiology, and End Results (SEER) database who had IPC (n = 524) or infiltrating ductal carcinoma (IDC) (n = 232,647). Generally, IPCs occurred in older women (≥50 years old) and presented with smaller sizes, lower grades, higher rates of oestrogen receptor (ER) and progesterone receptor (PR) positivity, and reduced lymph node (LN) involvement and were less likely to be treated with mastectomy than patients with IDC. The five-year disease-specific survival (DSS) rates were significantly better in IPC than in IDC (97.5% vs. 93%, respectively; P < 0.001). In the multivariate analysis, patients with IPC showed a DSS that was similar to that of IDC (hazard ratio = 0.556, 95% confidence interval 0.289–1.070, P = 0.079). No significant difference was observed in DSS between matched IPC and IDC groups (P = 0.085). Differences in outcomes may be partially explained by differences in tumour grade, LN status, and ER and PR status between the 2 groups. Gaining an improved clinical and biological understanding of IPC might result in more tailored and effective therapies in breast cancer patients.

Invasive papillary carcinoma (IPC) is defined as having papillary architecture in >90% of the invasive component^1^. Papillary carcinoma has been reported in most studies to include IPC and intraductal papillary carcinoma. The overall incidence of IPC is low, accounting for less than 1–2% of all newly diagnosed cases of invasive breast cancer^2^,^3^. Previous studies have revealed some of the characteristic properties of IPC. The majority of IPCs exhibit positive oestrogen receptor (ER) and progesterone receptor (PR) expression, while human epidermal growth factor receptor 2 (HER2) amplification is rarely observed^4^,^5^.

Although its rarity has prevented researchers from firmly defining the prognostic features of invasive papillary breast carcinoma, the data have suggested a more favourable clinical outcome for these patients than is observed in IDC patients^4^,^5^,^6^,^7^. Because of its low incidence, most of the available studies are case reports or small retrospective studies, and very few are series studies. Mitnick *et al*.^8^ reported that the 5-year disease-free survival rate of IPC is approximately 90%, and Schneider *et al*.^9^ reported that the 10-year survival rate is 86%. Vural O *et al*.^5^ analysed 24 cases of IPC with overall favourable prognoses. Berg J W *et al*.^6^ identified 1364 papillary adenocarcinoma patients in the Surveillance, Epidemiology, and End Results (SEER) registries from 1973–1987 and reported that IPC has a 5-year relative survival rate of 95%, while IDC has a 5-year relative survival rate of 79%. According to an analysis from the Netherlands Cancer Registry, in which 1078 patients with papillary tumours were enrolled from 1989 to 2003, patients with papillary carcinoma have prolonged survival, but with a wide confidence interval (odds ratio for survival = 0.57; 95% confidence interval [95% CI], 0.2–1.6)^7^. However, that study may have included misclassification bias because IPC may not have been clearly classified before 2003^10^. Moreover, distinguishing between invasive and non-invasive forms of papillary carcinoma is critical because each has a unique prognosis. Large series, however, often include these diagnoses as an amalgam because of their relatively low cumulative frequency^6^,^7^, and this may result in the presence of confounding factors when characterizing IPC.

Limited data are available that contribute to a comprehensive summarization of the clinicopathological characteristics and prognostic factors that are associated with IPC. And the prognostic values of demographic and clinicopathological characteristics in IPC therefore remains unclear. The data presented by Berg J W *et al*. lacked tumour clinicopathological characteristics^6^. In a study by Vural O *et al*. grade 1 tumours comprised only 16.6% of the total, and their sample size was too small to conduct a multivariate analysis^5^. In Louwman’s study, the grade 1 tumours comprised only 17% of the total, but 59% of the tumours lacked grade information, and hormone receptor information was not included^7^. Liu *et al*.^4^ conducted a survival analysis in which they compared IPC and IDC groups that were randomly matched by age, menopausal status, LN status, tumour size and tumour grade. However, the matched variables did not include ER or PR status, and the IPC sample size was only 83. Previous studies have often lacked adequate follow-up, a detailed description of clinical characteristics, adjustments for confounding factors and adequate sample sizes. Outside of analyses of large registries, which include multiple rare tumour types, a limited number of studies have focused solely on IPC. In the absence of definitive guidelines for its management, IPC treatments are currently based on evidence from studies of IDC, which may be inappropriate. Identifying the prognostic factors of IPC would help physicians to acquire a better understanding of the disease and make better informed therapeutic decisions. It is therefore of great importance to clarify the clinicopathological characteristics and prognostic factors of IPC in a large population.

This study used data from the SEER dataset, which is a large United States population-based cancer registry, to determine and compare survival outcomes in patients with IPC and IDC. We sought to determine the prognostic factors that may account for survival differences between these two histologic subtypes of breast cancer.

## Results

### Clinicopathological Characteristics of IPC

Overall, 233,171 patients with breast cancer were enrolled, including 524 IPC patients and 232,647 IDC patients. The demographics and tumour and treatment characteristics of IPC were compared to those of IDC, and the results are summarized in Table 1. There were considerable differences in tumour characteristics, including histological grade, tumour size, LN status, AJCC stage, ER status, PR status and HER2 status, between the 2 populations. IPC patients presented with smaller tumours (tumour size <20 mm: 67.4% vs. 63.9%, respectively; P = 0.013) and more grade 1 disease (32.6% vs. 18.6%, respectively; P < 0.001) than were observed in IDC patients. Furthermore, the rate of LN involvement at diagnosis was lower in IPC patients than in IDC patients (11.6% vs. 32.6%, respectively; P < 0.001). IPC patients may more frequently present with AJCC stage I disease than IDC patients (61.5% vs. 50.2%, respectively; P < 0.001). ER positivity was detected in 87.2% of IPCs and 76.6% of IDCs (P < 0.001). Similarly, PR was expressed in 80.7% of IPCs and 66.5% of IDCs (P < 0.001). Table 1 and Supplementary Table S1 outline the results for HER2 amplification status. HER2 positivity was lower in IPC than in IDC (Table 1: 2.1% vs. 5.6%, respectively; P < 0.001 and Supplementary Table S1: 2.0% vs. 4.8%, respectively; P = 0.006). Treatments were also different between the groups. Lumpectomy rates were higher in IPC than in IDC (68.7% vs. 60.2%, respectively; P < 0.001), and adjuvant radiation was used less frequently in IPC than IDC (48.5% vs. 56.6%, respectively; P = 0.001).

### Comparison of Survival between IPCs and IDCs

**Figure 1:**
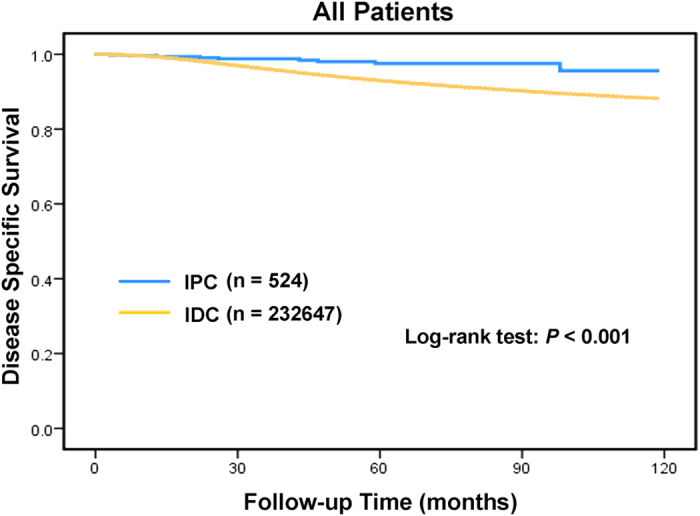
Log-rank test for breast cancer disease-specific survival to compare invasive papillary carcinoma (IPC) to infiltrating ductal carcinoma (IDC): χ^2^ = 12.631, P < 0.001.

As shown in Kaplan–Meier plots, disease-specific survival (DSS) was better in IPC patients than in the overall IDC population (χ^2^ = 12.631, P < 0.001, Fig. 1). The five-year DSS rates in IPC and IDC were 97.5% (95% CI: 99.3–95.7%) and 93% (95% CI: 92.7–93.1%), respectively. A Cox proportional hazards model was used to investigate the effects of baseline characteristics on DSS (Table 2). Prognostic indicators were found to be significantly associated with DSS in the univariate analysis. These included the year of diagnosis, age, race, marital status, laterality, tumour grade, tumour size, LN status, ER status, PR status, HER2 status, radiation and surgery type (Table 2). IPC histology was found to be a protective factor (hazard ratio [HR] = 0.325, 95% CI 0.169–0.625, P < 0.001). All of these variables were therefore included in the multivariate analysis, which confirmed the prognostic factors that were identified in the univariate analysis (Table 2). However, after adjusting for other prognostic factors, histological type was no longer an independent prognostic factor in the multivariate analysis (HR = 0.556, 95% CI 0.289–1.070, P = 0.079).

### Survival Analysis in Matched Groups

**Figure 2:**
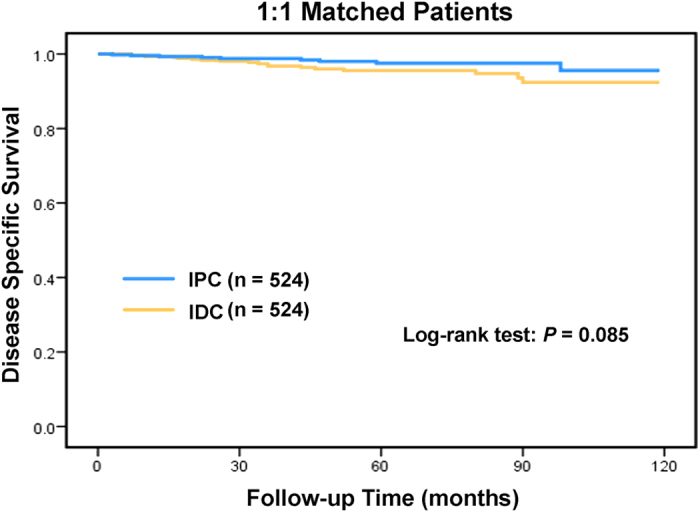
Log-rank test of 1:1 matched groups to compare invasive papillary carcinoma (IPC) to infiltrating ductal carcinoma (IDC): χ^2^ = 2.976, P = 0.085.

To ensure that differences in outcomes were not based on baseline differences in demographic and clinical characteristics across histologic subtypes, we performed a 1:1 (IPC: IDC) matched case-control analysis using the propensity score-matching method. We obtained a group of 1048 patients, including 524 patients with each histological type (Table 3). There was no significant difference in characteristics between IPC and IDC in the matched groups. Furthermore, we found that IPC histology was no longer associated with a better prognosis in DSS (χ^2^ = 2.976, P = 0.085, Fig. 2).

### Baseline Characteristics and Survival Outcomes in the ER-Positive Subgroup

Most IPCs are ER-positive tumours. When the analysis was limited to 178,755 ER-positive IPC and IDC patients (457 IPCs and 178,298 IDCs), similar results were observed (Supplementary Table S1). Specifically, ER-positive IPC patients had smaller tumours with lower grades, lower AJCC stages, lower LN-positivity, higher PR-positivity and a higher lumpectomy rate than ER-positive IDC patients. This comparison showed nearly the same curves as the analysis that was described above for all patients (See Supplementary Fig. S1). Within the ER-positive subset, patients with IPC had a better DSS than patients with IDC (P = 0.004).

### Subgroup Analyses

**Figure 3:**
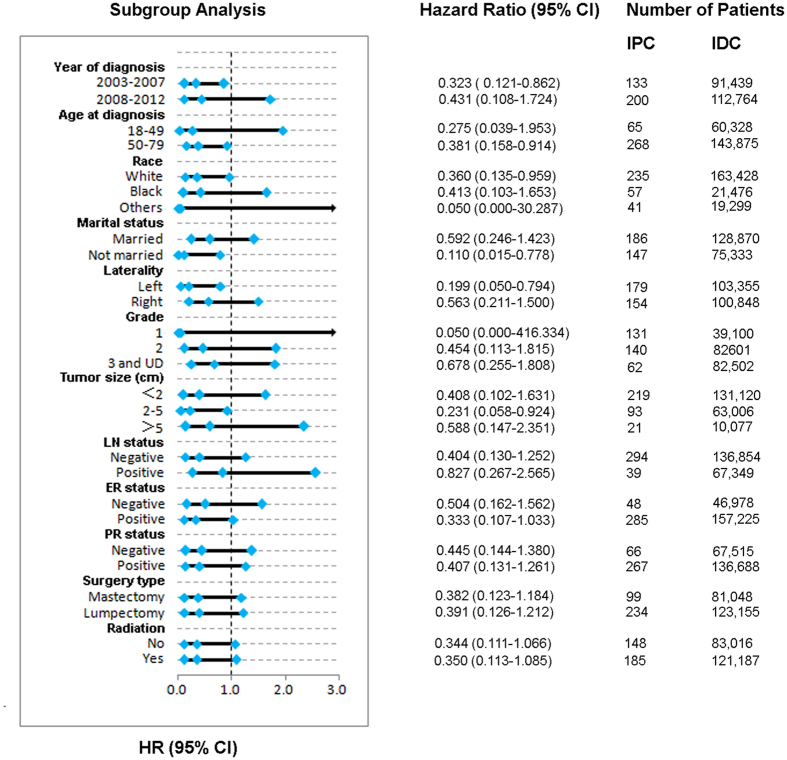
Forest plot of hazard ratios (HRs) for invasive papillary carcinoma (IPC) versus infiltrating ductal carcinoma (IDC) in the subgroup analysis. The diamond on the X-axis indicates the HR and the 95% confident interval (CI) of each subgroup.

A forest plot of HRs that was used to illustrate the exploratory subgroup analyses suggested that in some subgroups, an IPC subtype was no longer a positive prognostic indicator for DSS (Fig. 3). HRs in different tumour grade subgroups were not significantly different between IPC and IDC (grade 1: HR = 0.05, 95% CI 0.00–416.33, P = 0.515; grade 2: HR = 0.454, 95% CI 0.113–1.815, P = 0.264; and grade 3 and undifferentiated: HR = 0.678, 95% CI 0.255–1.808, P = 0.438). Moreover, HRs in different LN status subgroups were also not significantly different between IPC and IDC (negative: HR = 0.404, 95% CI 0.130–1.252, P = 0.116; and positive: HR = 0.827, 95% CI 0.267–2.565, P = 0.742). The HRs in different ER and PR status subgroups were similar (ER-negative: HR = 0.504, 95% CI 0.162–1.562, P = 0.235; ER-positive: HR = 0.333, 95% CI 0.107–1.033, P = 0.057; PR-negative: HR = 0.445, 95% CI 0.144–1.380, P = 0.161; and PR-positive: HR = 0.407, 95% CI 0.131–1.261, P = 0.119). These results suggest that tumour grade, LN status, and ER and PR status may be principal confounders in IPC prognoses.

## Discussion

As the incidence of breast cancer increases, the incidence and the number of patients with rare histological subtypes may also increase. Therefore, it is desirable to gain more knowledge regarding the clinical and biological features of IPC. A large population is needed to obtain a sufficient number of patients with these relatively rare tumours within a reasonable timespan. In this study, we retrospectively investigated the clinicopathological characteristics and survival outcomes of IPC in a large population. Our findings indicate that IPCs have unique pathological characteristics, are more likely to be treated with breast-conserving surgery and are associated with more favourable prognoses than are IDCs in DSS. After adjusting for confounding factors, IPC patients did not, however, have a significant survival advantage over IDC patients. Further subgroup analyses revealed that the differences in the distributions of tumour grade, LN status, and ER and PR status may account for the improved survival observed in IPC.

This study is currently the largest analysis of IPC. We summarized the clinicopathological characteristics of IPC and found that this specific histological type was associated with a lower grade, a smaller tumour size, reduced LN involvement, earlier stages, higher hormone expression positivity, and lower HER2 amplification rates than were IDCs. Some of these results are in agreement with those in previous studies^4^,^5^,^9^. In a univariate analysis, survival was significantly better in IPC than in IDC, which is consistent with previous studies^5^,^6^,^7^. However, after adjusting for potential confounders in a multivariate Cox regression analysis, we found that the survival advantage in IPC disappeared. Furthermore, after matching IPC and IDC 1:1 by year of diagnosis, age, race, marital status, laterality, tumour grade, tumour size, LN status, tumour stage, ER status, PR status, HER2 status and surgery type, we found that IPC and IDC patients had nearly the same DSS. Collectively, these results imply that the IPC-specific histological type is not an independent prognostic factor. To identify the underlying factors that contributed to this phenomenon, we performed subgroup analyses. The results from the subgroup analyses showed that no prognostic superiority was observed for IPC in tumour grade, LN status, ER status or PR status subgroups, suggesting that the differences observed in survival outcomes between IPC and IDC resulted primarily from the distributions of tumour grades, LN statuses, and ER and PR statuses in the 2 tumour types.

Only a limited amount of information about tumour clinicopathological characteristics has been reported in previous studies. Analyses have sporadically referred to the prognostic values of tumour characteristics in IPC. Liu’s study^4^ demonstrated the prognostic value of LN status and molecular subtype in IPC. Louwman *et al*. observed better age-, stage- and grade-adjusted prognoses in patients with lobular, mucinous, medullary, and tubular tumours but not papillary or cribriform tumours^7^. However, none of these studies have systematically and convincingly indicated a dominant prognostic value for tumour grade, LN status or ER and PR status in IPCs. The results of our subgroup and matched comparison analyses support the hypothesis that tumour grade, LN status, and ER and PR status are primary prognostic factors in the IPC subtype. The underlying mechanisms that contribute to the prognostic values of tumour grade and hormone receptor status may include the following. Subjectively, histologic grades are assessed as a composite of tubular differentiation, nuclear features, and mitotic activity, and they are an important component when evaluating breast cancers and a required parameter in pathological reports of breast cancers^11^. A lower grade indicates a cancer with a tubular structure, reduced nuclear pleomorphism and reduced mitosis and a carcinoma with less invasive biological behaviours. It is generally accepted that luminal cancers have a more favourable prognosis than other subtypes^12^,^13^. Furthermore, patients with luminal tumours benefit from adjuvant hormonal therapy, which is known to reduce local recurrence rates and mortality by 30%^14^. Loss of PR has been suggested as a marker for aberrant growth factor signalling and is associated with one mechanism for endocrine resistance^15^.

The results of this study have several therapeutic implications. Because histological type was not an independent prognostic factor in the multivariate analysis, therapeutic decisions should not be made based solely on this rare entity. Further analysis according to the propensity score matching method ensured the establishment of well-balanced baseline characteristics for the two histological groups, and this method again proved the above point. Therefore, treatment should not be lessened for IPC patients who are similar to IDC patients in other clinicopathological characteristics. Moreover, because the subgroup analyses suggested that tumour grade, LN status, and ER and PR status are the predominant factors that caused the difference in survival between IPC and IDC groups, clinicians might need to take into account these prognostic indicators instead of histological types. For instance, in patients with high grade, ER-negative, PR-negative and LN-positive IPC, a treatment strategy should probably resemble the strategy used for IDC patients to ensure adequate therapeutic strength.

Our study has several limitations. First, records for Ki-67 expression, adjuvant chemotherapy and endocrine therapy were not available in the SEER database, which conceals important prognostic factors from researchers. However, the absence of data for chemotherapy does not significantly affect our findings because currently, the use of chemotherapy in breast cancer is based on disease stage and molecular subtype and not on histologic subtype. Second, we used the propensity score method for matching. In this procedure, 524 IDCs were matched with IPCs that were selected randomly from the patient population, and this may have caused sampling bias and decreased the external validity of our data.

We investigated a large cohort of patients with IPC and found that this rare tumour type presents unique clinicopathological characteristics and is associated with a higher rate of breast-conserving surgery and favourable prognoses than are observed in the overall IDC population. However, this advantage was diminished after we adjusted for demographic and clinicopathological factors. Therefore, patients diagnosed with this rare variant should be made aware that its biological features are not as favourable as once thought. Practitioners should continue to strictly follow evidence-based treatment guidelines, and further validation of these results in a large population may help to clarify this issue. Improving our understanding of the clinical and biological features of IPC may lead to more individualized and tailored therapies for breast cancer patients.

## Methods

### Ethics Statement

We obtained permission to access the SEER research data files using the reference number 13487-Nov2014. The data released by the SEER database do not require informed patient consent, and our study was approved by the Ethical Committee and Institutional Review Board of Fudan University Shanghai Cancer Centre (FDUSCC). The methods were performed in accordance with the approved guidelines.

### Data Acquisition and Patient Selection

We used the SEER dataset that was released in April 2015, which included data from 18 population-based registries (1973–2012) and covered approximately 28% of U.S. cancer patients. Data for tumour location, grade, and histology were recorded according to the International Classification of Diseases for Oncology Version 3 (ICD-O-3). The inclusion criteria used to identify eligible patients were the following: females aged between 18 and 79, unilateral breast cancer, breast cancer (ICD-O-3 site code C50) as the first and only cancer diagnosis, diagnosis not obtained from a death certificate or autopsy, only one primary site, pathological confirmation of infiltrating ductal carcinoma, not otherwise specified (IDC-NOS) (ICD-O-3 8500/3) and papillary carcinoma (ICD-O-3 8050/3) with invasion (behaviour code ICD-O-3 malignant), surgical treatment with either mastectomy, breast-conserving surgery or unknown type, known ER and PR statuses, American Joint Committee on Cancer (AJCC) stages I–III, and known time of diagnosis from January 1, 2003 to December 31, 2012. Patients diagnosed with breast cancer before 2003 were excluded because the World Health Organization (WHO) did not recognize IPC as a distinct pathological entity until 2003. In addition, patients who were diagnosed with breast cancer after 2012 were not included because the database was only updated up to December 31, 2012, and we wanted to ensure adequate follow-up time. A total of 233,171 patients were included. Of these patients, 524 were diagnosed with IPC, and 232,647 were diagnosed with IDC.

The collected demographic statistics included the year of diagnosis, age at diagnosis, race, and marital status. We treated age at diagnosis as a binary variable that was classified using the following age groups: 18 to 49 years old and 50 to 79 years old. Tumour characteristics included laterality, histologic grade, tumour size, regional LN status, AJCC stage, ER status, PR status, and HER2 status. Among these variables, tumour size was treated as a categorical variable as follows: <20 mm, 20 to 50 mm, and >50 mm. For HER2 status, data were available only after 2010 for both subtypes as a result of the limitations of the SEER dataset.

### Outcome Measurement

In the present study, DSS was used as the primary study outcome and was calculated from the date of diagnosis to the date of death caused by breast cancer. Patients who died from other causes unrelated to a breast cancer diagnosis or who were alive were censored on the date of death or the date of last contact.

The study cut-off date was predetermined by the SEER November 2014 submission databases, which contained complete death data through 2012. Therefore, December 31, 2012 was the study cut-off date. The following algorithms were used in the SEER databases: date of last contact = min (date of last contact, study cut-off date), and survival months = floor ((date of last contact–date dx)/days in a month).

### Statistical Analysis

Clinicopathological characteristics were compared across groups using Pearson’s Chi-square tests or Fisher’s exact tests for categorical nominal data and Cochran-Mantel Haenszel (CMH) Chi-square tests for categorical ordinal data. Survival curves were generated using the Kaplan-Meier method, and differences between curves were analysed using log-rank tests. Univariate and multivariate Cox proportional hazard models were applied to identify factors that are associated with DSS, and HRs and 95% CIs were reported.

To account for differences in baseline characteristics across groups, we matched each IPC patient to 1 IDC patient using the following predetermined factors: year of diagnosis, age, race, marital status, laterality, tumour grade, tumour size, LN status, tumour stage, ER status, PR status, HER2 status and surgery type. We used psmatching3 in SPSS, which was designed for the propensity score matching method and to test the matching quality to determine the balance after the match. Because the majority of IPC cases showed an ER-positive (ER+) status, a planned secondary survival comparison within ER+ patients was also conducted. Subgroup analyses using univariate Cox proportional hazard modelling evaluated the HRs of IPC versus IDC, and a forest plot was constructed to better present each prognostic factor’s effect on DSS.

All of the statistical analyses were performed using SPSS statistical software, version 19.0 (IBM Corp, Armonk, NY). A two-sided P < 0.05 was considered to indicate statistical significance.

## Additional Information

**How to cite this article**: Zheng, Y.-Z. *et al*. Clinicopathological Characteristics and Survival Outcomes in Invasive Papillary Carcinoma of the Breast: A SEER Population-Based Study. *Sci. Rep.* **6**, 24037; doi: 10.1038/srep24037 (2016).

## Supplementary Material

Supplementary Information

## Figures and Tables

**Table 1:** Patient Characteristics in IPC Compared to IDC^a^.

	IPC, n = 524 (%)	IDC, n = 232647 (%)	Total, n = 233171 (%)	*P*-Value^b^
Median follow-up (months) (IQR)	43.5 (17–75.8)	46 (21–76)	46 (21–76)	
Year of diagnosis				
2003–2007	216 (41.2)	102520 (44.1)	102736 (44.1)	0.190
2008–2012	308 (58.8)	130127 (55.9)	130435 (55.9)	
Age at diagnosis (years)				
18–49	92 (17.6)	68569 (29.5)	68661 (29.4)	<0.001
50–79	432 (82.4)	164078 (70.5)	164510 (70.6)	
Race				
White	366 (69.8)	184589 (79.3)	184955 (79.3)	<0.001
Black	86 (16.4)	25157 (10.8)	25243 (10.8)	
Others^c^	68 (13.0)	21658 (9.3)	21726 (9.3)	
Unknown	4 (0.8)	1243 (0.5)	1247 (0.5)	
Marital status				
Married	261 (49.8)	140697 (60.5)	140958 (60.5)	<0.001
Not married^d^	232 (44.3)	83290 (35.8)	83522 (35.8)	
Unknown	31 (5.9)	8660 (3.7)	8691 (3.7)	
Laterality				
Left	275 (52.5)	117898 (50.7)	118173 (50.7)	0.693
Right	249 (47.5)	114724 (49.3)	114973 (49.3)	
Only one side, NOS	0 (0.0)	25 (0.0)	25 (0.0)	
Grade				
1	171 (32.6)	43167 (18.6)	43338 (18.6)	<0.001
2	167 (31.9)	91394 (39.3)	91561 (39.3)	
3 and UD^e^	76 (14.5)	92032 (39.6)	92108 (39.5)	
Unknown	110 (21.0)	6054 (2.6)	6164 (2.6)	
Tumour size (cm)				
<2	353 (67.4)	148588 (63.9)	148941 (63.9)	0.013
2–5	133 (25.4)	71128 (30.6)	71261 (30.6)	
>5	37 (7.1)	11768 (5.1)	11805 (5.1)	
Unknown	1 (0.2)	1163 (0.5)	1164 (0.5)	
LN status				
Negative	400 (76.3)	150799 (64.8)	151199 (64.8)	<0.001
Positive	61 (11.6)	75819 (32.6)	75880 (32.5)	
Unknown	63 (12.0)	6029 (2.6)	6092 (2.6)	
AJCC stage				
I	322 (61.5)	116731 (50.2)	117053 (50.2)	<0.001
II	169 (32.3)	86931 (37.4)	87100 (37.4)	
III	33 (6.3)	28985 (12.5)	29018 (12.4)	
ER status				
Negative	67 (12.8)	54349 (23.4)	54416 (23.3)	<0.001
Positive	457 (87.2)	178298 (76.6)	178755 (76.7)	
PR status				
Negative	101 (19.3)	77918 (33.5)	78019 (33.5)	<0.001
Positive	423 (80.7)	154729 (66.5)	155152 (66.5)	
HER2 status				
Negative	171 (32.6)	63553 (27.3)	63724 (27.3)	<0.001
Positive	11 (2.1)	12918 (5.6)	12929 (5.5)	
Borderline	2 (0.4)	1859 (0.8)	1861 (0.8)	
Unknown	340 (64.9)	154317 (66.3)	154657 (66.3)	
Total	524 (100.0)	232647 (100.0)	233171 (100.0)	
Surgery type				
Mastectomy	164 (31.3)	92247 (39.7)	92411 (39.6)	<0.001
Lumpectomy	360 (68.7)	140144 (60.2)	140504 (60.3)	
Unknown	0 (0.0)	256 (0.1)	256 (0.1)	
Radiation				
No	248 (47.3)	93361 (40.1)	93609 (40.1)	0.001
Yes	254(48.5)	131620 (56.6)	131874 (56.6)	
Unknown	22 (4.2)	7666 (3.3)	7688 (3.3)	

AJCC = American Joint Committee on Cancer, ER = oestrogen receptor, HER2 = human epidermal growth factor receptor 2, IPC = invasive papillary carcinoma, IDC = infiltrating ductal carcinoma, IQR = interquartile range, LN = lymph node, NOS = no other specific, PR = progesterone receptor, UD = undifferentiated.

^a^The data are presented as the No.(percentage) of patients unless otherwise indicated.

^b^*P*-value of the Chi-square test to compare the IPC and IDC groups.

^c^Including American Indian/Alaskan native, Asian/Pacific Islander and others-unspecified.

^d^Including divorced, separated, single (never married) and widowed.

^e^Including grade 3 and undifferentiated.

**Table 2:** Univariate and Multivariate Analysis of Disease-specific Survival (DSS).

Variables	Univariate analysis		Multivariate analysis	
	HR (95% CI)	*P*-Value	HR (95% CI)	*P*-Value
Year of diagnosis				
2003–2007	Reference	–	Reference	–
2008–2012	0.793 (0.759–0.827)	<0.001	0.856 (0.816–0.897)	<0.001
Age at diagnosis (years)				
18–49	Reference	–	Reference	–
50–79	0.866 (0.835–0.898)	<0.001	1.205 (1.162–1.250)	<0.001
Race				
White	Reference	–	Reference	–
Black	2.043 (1.955–2.135)	<0.001	1.267 (1.211–1.326)	<0.001
Others^a^	0.863 (0.807–0.923)	<0.001	0.796 (0.744–0.851)	<0.001
Unknown	0.404 (0.263–0.620)	<0.001	0.374 (0.244–0.574)	<0.001
Marital status				
Married	Reference	–	Reference	–
Not married^b^	1.428 (1.378–1.478)	<0.001	1.243 (1.199–1.288)	<0.001
Unknown	1.052 (0.946–1.170)	0.353	1.019 (0.916–1.133)	0.734
Laterality				
Left	Reference	–	Reference	–
Right	0.950 (0.918–0.983)	0.003	0.956 (0.924–0.989)	0.010
Only one side, NOS	0.788 (0.111–5.591)	0.811	0.514 (0.072–3.651)	0.506
Grade				
1	Reference	–	Reference	–
2	3.709 (3.337–4.123)	<0.001	2.274 (2.044–2.530)	<0.001
3 and UD^c^	11.479 (10.369–12.707)	<0.001	3.745 (3.368–4.163)	<0.001
Unknown	5.006 (4.280–5.856)	<0.001	2.323 (1.982–2.722)	<0.001
Histology type				
IDC	Reference	–	Reference	–
IPC	0.325 (0.169–0.625)	0.001	0.556 (0.289–1.070)	0.079
Tumour size (cm)				
<2	Reference	–	Reference	–
2–5	4.174 (4.008–4.346)	<0.001	2.211 (2.118–2.308)	<0.001
>5	10.272 (9.752–10.818)	<0.001	4.035 (3.809–4.274)	<0.001
Unknown	14.930 (13.424–16.606)	<0.001	5.525 (4.950–6.167)	<0.001
LN status				
Negative	Reference	–	Reference	–
Positive	4.354 (4.193–4.521)	<0.001	2.753 (2.645–2.865)	<0.001
Unknown	3.882 (3.554–4.240)	<0.001	3.347 (3.060–3.662)	<0.001
ER status				
Negative	Reference	–	Reference	–
Positive	0.286 (0.277–0.296)	<0.001	0.664 (0.630–0.699)	<0.001
PR status				
Negative	Reference	–	Reference	–
Positive	0.296 (0.286–0.307)	<0.001	0.623 (0.592–0.657)	<0.001
HER2 status				
Negative	Reference	–	Reference	–
Positive	0.832 (0.691–1.003)	0.053	0.484 (0.402–0.583)	<0.001
Borderline	1.095 (0.741–1.619)	0.648	1.008 (0.682–1.491)	0.967
Unknown	1.234 (1.142–1.332)	0.000	0.922 (0.848–1.003)	0.060
Surgery type				
Mastectomy	Reference	–	Reference	–
Lumpectomy	0.392 (0.378–0.406)	<0.001	0.789 (0.758–0.822)	<0.001
Unknown	2.355 (1.719–3.225)	<0.001	1.502 (1.092–2.067)	0.012
Radiation				
No	Reference	–	Reference	–
Yes	0.739 (0.713–0.765)	<0.001	0.853 (0.821–0.886)	<0.001
Unknown	1.202 (1.097–1.318)	<0.001	1.040 (0.948–1.141)	0.408

CI = confidence interval, ER = oestrogen receptor, HER2 = human epidermal growth factor receptor 2, HR = hazard ratio, IPC = invasive papillary carcinoma, IDC = infiltrating ductal carcinoma, LN = lymph node, PR = progesterone receptor, UD = undifferentiated. Multivariate analysis included year of diagnosis, age at diagnosis, race, marital status, laterality, grade, histology, tumour size, LN status, ER status, PR status, HER2 status, surgery type and radiation.

^a^Including American Indian, Alaska Native, Asian, Pacific Islander and others-unspecified.

^b^Including divorced, separated, single (never married) and widowed.

^c^Including grade 3 and undifferentiated.

**Table 3:** Patient Characteristics in IPC Compared to IDC^a^ in 1:1 Matched Groups.

	IPC, n = 524 (%)	IDC, n = 524 (%)	Total, n = 1048 (%)	*P*-Value^b^
Median follow-up (months) (IQR)	43.5 (17–75.8)	42.5 (16.3–75.5)	43 (17–75)	
Year of diagnosis				
2003–2007	216 (41.2)	210 (40.1)	426 (40.6)	0.706
2008–2012	308 (58.8)	314 (59.9)	622 (59.4)	
Age at diagnosis (years)				
18–49	92 (17.6)	94 (17.9)	186 (17.7)	0.872
50–79	432 (82.4)	430 (82.1)	862 (82.3)	
Race				
White	366 (69.8)	376 (71.8)	742 (70.8)	0.880
Black	86 (16.4)	81 (15.5)	167 (15.9)	
Others^c^	68 (13.0)	62 (11.8)	130 (12.4)	
Unknown	4 (0.8)	5 (1.0)	9 (0.9)	
Marital status				
Married	261 (49.8)	268 (51.1)	529 (50.5)	0.905
Not married^d^	232 (44.3)	225 (42.9)	457 (43.6)	
Unknown	31 (5.9)	31 (5.9)	62 (5.9)	
Laterality				
Left	275 (52.5)	287 (54.8)	562 (53.6)	0.457
Right	249 (47.5)	237 (45.2)	486 (46.4)	
Grade				
1	171 (32.6)	159 (30.3)	330 (31.5)	0.529
2	167 (31.9)	170 (32.4)	337 (32.2)	
3 and UD^e^	76 (14.5)	92 (17.6)	168 (16.0)	
Unknown	110 (21.0)	103 (19.7)	213 (20.3)	
Tumour size (cm)				
<2	353 (67.4)	360 (68.7)	713 (68.0)	0.058
2–5	133 (25.4)	136 (26.0)	269 (25.7)	
>5	37 (7.1)	22 (4.2)	59 (5.6)	
Unknown	1 (0.2)	6 (1.1)	7 (0.7)	
LN status				
Negative	400 (76.3)	396 (75.6)	796 (76.0)	0.951
Positive	61 (11.6)	64 (12.2)	125 (11.9)	
Unknown	63 (12.0)	64 (12.2)	127 (12.1)	
AJCC stage				
I	332 (61.5)	328 (62.6)	650 (62.0)	0.791
II	169 (32.3)	168 (32.1)	337 (32.2)	
III	33 (6.3)	28 (5.3)	61 (5.8)	
ER status				
Negative	67 (12.8)	68 (13.0)	135 (12.9)	0.927
Positive	457 (87.2)	456 (87.0)	913 (87.1)	
PR status				
Negative	101 (19.3)	103 (19.7)	204 (19.5)	0.876
Positive	423 (80.7)	421 (80.3)	844 (80.5)	
HER2 status				
Negative	171 (32.6)	166 (31.7)	337 (32.2)	0.888
Positive	11 (2.1)	14 (2.7)	25 (2.4)	
Borderline	2 (0.4)	3 (0.6)	5 (0.5)	
Unknown	340 (64.9)	341 (65.1)	681 (65.0)	
Surgery type				
Mastectomy	164 (31.3)	167 (31.9)	331 (31.6)	0.842
Lumpectomy	360 (68.7)	357 (68.1)	717 (68.4)	
Radiation				
No	248 (47.3)	240 (45.8)	488 (46.6)	0.747
Yes	254(48.5)	265 (50.6)	519 (49.5)	
Unknown	22 (4.2)	19 (3.6)	41 (3.9)	

AJCC = American Joint Committee on Cancer, ER = oestrogen receptor, HER2 = human epidermal growth factor receptor 2, IPC = invasive papillary carcinoma, IDC = infiltrating ductal carcinoma, IQR = interquartile range, LN = lymph node, NOS= no other specific, PR = progesterone receptor, UD = undifferentiated.

^a^The data are presented as the No.(percentage) of patients unless otherwise indicated.

^b^*P*-value of the Chi-square test to compare the IPC and IDC groups.

^c^Including American Indian/Alaskan native, Asian/Pacific Islander and others-unspecified.

^d^Including divorced, separated, single (never married) and widowed.

^e^Including grade 3 and undifferentiated.
