# Supplementary material for: Clinicopathological Characteristics and Survival Outcomes in Invasive Papillary Carcinoma of the Breast: A SEER Population-Based Study
Source: Sci Rep. 2016 Apr 7;6:24037. doi: 10.1038/srep24037 (PMC4823738; doi:10.1038/srep24037)
Supplement: Supplementary Information [file srep24037-s1.pdf]

Clinicopathological Characteristics and Survival Outcomes in Invasive Papillary Carcinoma of the Breast: A SEER Population-Based Study

Yi-Zi Zheng,[1,2] M.D.; Xin Hu,[1,*] Ph.D.; Zhi-Ming Shao,[1,2,3,*] M.D.

1 Department of Breast Surgery, Key Laboratory of Breast Cancer in Shanghai, Fudan University Shanghai Cancer Center, Fudan University, Shanghai 200032, China

2 Department of Oncology, Shanghai Medical College, Fudan University, Shanghai 200032, China

3 Institute of Biomedical Science, Fudan University, Shanghai 200032, China

* Address for correspondence and reprints:

Xin Hu, Ph.D.

Key Laboratory of Breast Cancer in Shanghai,

Fudan University Shanghai Cancer Center,

270 Dong'An Road, Shanghai, 200032, P.R. China

Phone: +86-21-64175590 (83423)

E-mail: xinhu.nlorg@gmail.com

Zhi-Ming Shao, M.D.

Department of Breast Surgery,

Fudan University Shanghai Cancer Center,

270 Dong'An Road, Shanghai, 200032, P.R. China

Phone: +86-21-64434556

E-mail: zhimingshao@yahoo.com

Supplementary Figure

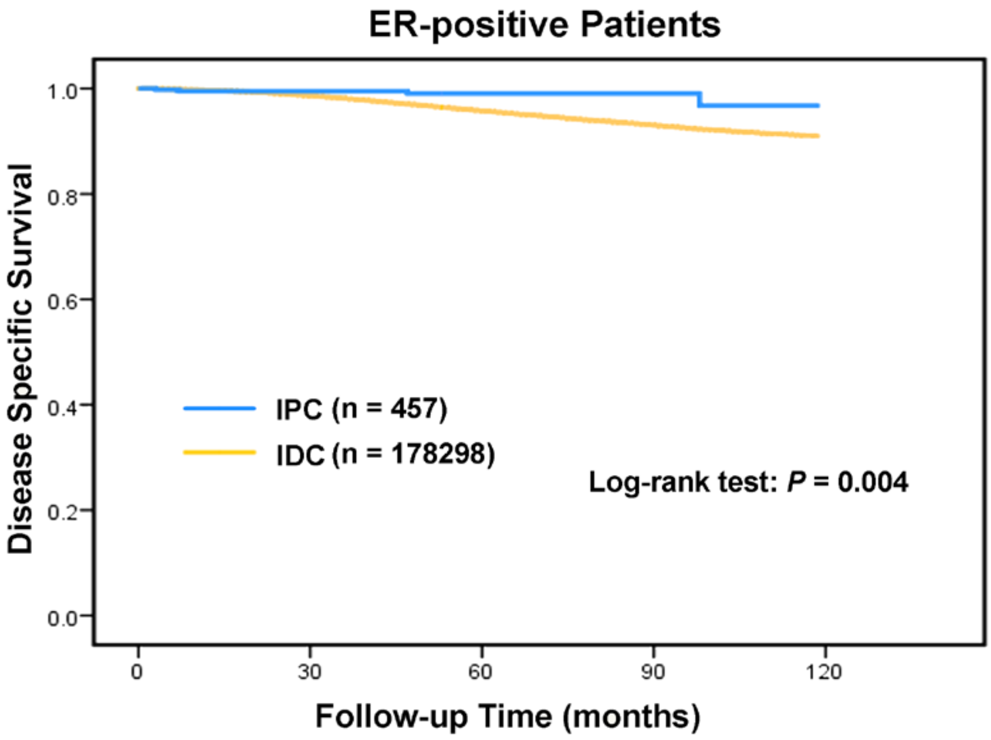

Figure S1: Log-rank test for breast cancer disease-specific survival in ER-positive patients to compare invasive papillary carcinoma (IPC) to infiltrating ductal carcinoma (IDC): $\chi^2 = 8.386$, P = 0.004.

Supplementary Table S1. Patient Characteristics in IPC Compared to IDC[a] in the ER-positive subgroup

	IPC, n = 457 (%)	IDC, n = 178298 (%)	Total, n = 178755 (%)	***P*-Value**[b]
Median follow-up (months) (IQR)	45 (17-76)	46 (21-76)	46 (21-76)	
Year of diagnosis				
2003-2007	184 (40.3)	75701 (42.5)	75885 (42.5)	0.343
2008-2012	273 (59.7)	102597 (57.5)	102870 (57.5)	
Age at diagnosis (years)				
18-49	65 (14.2)	49738 (27.9)	49803 (27.9)	$<$0.001
50-79	392 (85.8)	128560 (72.1)	128952 (72.1)	
Race				
White	327 (71.6)	144665 (81.1)	144992 (81.1)	$<$0.001
Black	67 (14.7)	15721 (8.8)	15788 (8.8)	
Others[c]	60 (13.1)	16925 (9.5)	16985 (9.5)	
Unknown	3 (0.7)	987 (0.6)	990 (0.6)	
Marital status				
Married	227 (49.7)	108093 (60.6)	108320 (60.6)	$<$0.001
Not married[d]	203 (44.4)	63522 (35.6)	63725 (35.6)	
Unknown	27 (5.9)	6683 (3.7)	6710 (3.8)	
Laterality				
Left	240 (52.5)	89810 (50.4)	90050 (50.4)	0.644
Right	217 (47.5)	88471 (49.6)	88688 (49.6)	
Only one side, NOS	0 (0.0)	17 (0.0)	17 (0.0)	
Grade				
1	169 (37.0)	42219 (23.7)	42388 (23.7)	$<$0.001
2	152 (33.3)	82376 (46.2)	82528 (46.2)	
3 and UD[e]	35 (7.7)	49392 (27.7)	49427 (27.7)	

Unknown	101 (22.1)	4311 (2.4)	4412 (2.5)	
Tumour size (cm)				
<2	326 (71.3)	122140 (68.5)	122466 (68.5)	0.004
2-5	102 (22.3)	48549 (27.2)	48651 (27.2)	
>5	29 (6.3)	6963 (3.9)	6992 (3.9)	
Unknown	0 (0.0)	646 (0.4)	646 (0.4)	
LN status				
Negative	352 (77.0)	117088 (65.7)	117440 (65.7)	<0.001
Positive	48 (10.5)	56594 (31.7)	56642 (31.7)	
Unknown	57 (12.5)	4616 (2.6)	4673 (2.6)	
AJCC stage				
I	298 (65.2)	96453 (54.1)	96751 (54.1)	<0.001
II	134 (29.3)	62606 (35.1)	62740 (35.1)	
III	25 (5.5)	19239 (10.8)	19264 (10.8)	
PR status				
Negative	37 (8.1)	26557 (14.9)	26594 (14.9)	<0.001
Positive	420 (91.9)	151741 (85.1)	152161 (85.1)	
HER2 status				
Negative	156 (34.1)	52494 (29.4)	52650 (29.5)	0.006
Positive	9 (2.0)	8606 (4.8)	8615 (4.8)	
Borderline	2 (0.4)	1573 (0.9)	1575 (0.9)	
Unknown	290 (63.5)	115625 (64.8)	115915 (64.8)	
Surgery type				
Mastectomy	135 (29.5)	66286 (37.2)	66421 (37.2)	0.003
Lumpectomy	322 (70.5)	111841 (62.7)	112163 (62.7)	
Unknown	0 (0.0)	171 (0.1)	171 (0.1)	
Radiation				
No	214 (46.8)	69126 (38.8)	69340 (38.8)	<0.001

Yes	223 (48.8)	103603 (58.1)	103826 (58.1)
Unknown	20 (4.4)	5569 (3.1)	5589 (3.1)

AJCC = American Joint Committee on Cancer, ER = oestrogen receptor, HER2 = human epidermal growth factor receptor 2, IPC = invasive papillary carcinoma, IDC = infiltrating ductal carcinoma, IQR = interquartile range, LN = lymph node, NOS= no other specific, PR = progesterone receptor, UD = undifferentiated.

[a]The data are presented as the No.(percentage) of patients unless otherwise indicated.

[b]*P*-value of the Chi-square test to compare the IPC and IDC groups.

[c] Including American Indian/Alaskan native, Asian/Pacific Islander and others-unspecified.

[d] Including divorced, separated, single (never married) and widowed.

[e] Including grade 3 and undifferentiated.
